# A Selected *Lactobacillus rhamnosus* Strain Promotes EGFR-Independent Akt Activation in an Enterotoxigenic *Escherichia coli* K88-Infected IPEC-J2 Cell Model

**DOI:** 10.1371/journal.pone.0125717

**Published:** 2015-04-27

**Authors:** Wei Zhang, Yao-Hong Zhu, Jin-Cai Yang, Gui-Yan Yang, Dong Zhou, Jiu-Feng Wang

**Affiliations:** Department of Veterinary Clinical Sciences, College of Veterinary Medicine, China Agricultural University, Beijing, China; Emory University School of Medicine, UNITED STATES

## Abstract

Enterotoxigenic *Escherichia coli* (ETEC) are important intestinal pathogens that cause diarrhea in humans and animals. Although probiotic bacteria may protect against ETEC-induced enteric infections, the underlying mechanisms are unknown. In this study, porcine intestinal epithelial J2 cells (IPEC-J2) were pre-incubated with and without *Lactobacillus rhamnosus* ATCC 7469 and then exposed to F4^+^ ETEC. Increases in *TLR4* and *NOD2* mRNA expression were observed at 3 h after F4^+^ ETEC challenge, but these increases were attenuated by *L*. *rhamnosus* treatment. Expression of *TLR2* and *NOD1* mRNA was up-regulated in cells pre-treated with *L*. *rhamnosus*. Pre-treatment with *L*. *rhamnosus* counteracted F4^+^ ETEC-induced increases in TNF-α concentration. Increased PGE_2_. concentrations were observed in cells infected with F4^+^ ETEC and in cells treated with *L*. *rhamnosus* only. A decrease in phosphorylated epidermal growth factor receptor (EGFR) was observed at 3 h after F4^+^ ETEC challenge in cells treated with *L*. *rhamnosus*. Pre-treatment with *L*. *rhamnosus* enhanced Akt phosphorylation and increased ZO-1 and occludin protein expression. Our findings suggest that *L*. *rhamnosus* protects intestinal epithelial cells from F4^+^ ETEC-induced damage, partly through the anti-inflammatory response involving synergism between TLR2 and NOD1. In addition, *L*. *rhamnosus* promotes EGFR-independent Akt activation, which may activate intestinal epithelial cells in response to bacterial infection, in turn increasing tight junction integrity and thus enhancing the barrier function and restricting pathogen invasion. Pre-incubation with *L*. *rhamnosus* was superior to co-incubation in reducing the adhesion of F4^+^ ETEC to IPEC-J2 cells and subsequently attenuating F4^+^ ETEC-induced mucin layer destruction and suppressing apoptosis. Our data indicate that a selected *L*. *rhamnosus* strain interacts with porcine intestinal epithelial cells to maintain the epithelial barrier and promote intestinal epithelial cell activation in response to bacterial infection, thus protecting cells from the deleterious effects of F4^+^ ETEC.

## Introduction

Enterotoxigenic *Escherichia coli* (ETEC) strains are not only the most common cause of travelers’ diarrhea, which can be fatal for children under 5 years of age, they are also the leading cause of post-weaning diarrhea (PWD) in piglets [1,2]. The most prevalent ETEC strain implicated in PWD in piglets expresses F4 (K88)^+^ fimbriae. Our previous studies have shown that administration of *Lactobacillus rhamnosus* ameliorates F4^+^ ETEC-induced diarrhea in newly weaned piglets; however, pre-treatment with high doses of *L*. *rhamnosus* may negate the preventative effects [3–5]. Accumulating evidence suggests that the beneficial effects of *Lactobacillus* strains may be due to their ability to restore the normal microbiota, inhibit pathogen adhesion to the intestinal wall, and maintain the membrane barrier [6–8]. However, the exact mode of action of lactobacilli in this regard remains largely unknown.

Intestinal epithelial cells (IECs) comprise the largest and most important anatomic as well as immunologic barrier against external environmental stimuli. The mucus layer coating the IECs serves as the first line of intestinal defense against infection by physically protecting the cells from direct exposure to bacteria and other antigens [9]. ETEC are capable of gaining access to enterocytes in the small intestine through EatA-induced degradation of MUC2 [10].

Two types of pattern recognition receptors (PRRs), the membrane-bound Toll-like receptors (TLRs) and the cytoplasmic Nod-like receptors (NLRs), provide complementary pathogen surveillance [11]. In general, binding of pathogens to TLRs or NLRs activates nuclear factor-κB (NF-κB) signaling and leads to the production of pro-inflammatory cytokines, chemokines, and antimicrobial peptides, thereby contributing to host defense and inflammation [12]. In addition, various PRRs are involved in regulating intestinal epithelial barrier integrity. Lipopolysaccharide (LPS) increases intestinal tight junction (TJ) permeability both *in vitro* and *in vivo* by inducing enterocyte membrane expression of TLR4 and CD14 [13]. Activation of the phosphatidylinositol-3-kinase (PI3K) pathway as a result of TLR2 signaling strengthens the TJ-associated epithelial barrier [14]. To date, knowledge regarding the mechanism underlying probiotic modulation of the intestinal barrier remains limited, however.

The epithelium maintains its selective barrier function through TJs that mechanically link adjacent cells and seal the intercellular space. The primary proteins thus far identified as TJ-specific integral transmembrane proteins include occludin and the claudins. In addition, the zonula occludens (ZO) may act as a link between the cytoskeleton and other TJ proteins [15]. It has been shown that *L*. *rhamnosus* GG (LGG, ATCC 53103) promotes expression of ZO-1 and occludin in Caco-2 cells [8,16]. In a piglet diarrhea model, *L*. *plantarum* inhibited ETEC K88-induced decreases in occludin mRNA and protein levels in the jejunum [17].

Epidermal growth factor receptor (EGFR) signaling is involved in regulating cellular proliferation, differentiation, and survival. Ligation of EGFR by its soluble ligands (EGF, heparin-binding-EGF, transforming growth factor, or amphiregulin) triggers the formation of homo- and hetero-dimers with other ErbB family members and the tyrosine auto-phosphorylation of several cytoplasmic proteins [18]. The indirect recruitment of PI3K to tyrosine-phosphorylated EGFR activates the downstream target Akt [19]. A recent study showed that the probiotic LGG transactivates EGFR, leading to suppression of apoptosis of mouse IECs induced by the cytokines TNF-α, IL-1α, and IFN-γ [20]. In a mouse model of colitis induced by 2,4,6-trinitrobenzene sulfonic acid, hirsutenone-mediated prevention of down-regulation of ZO-1 and occludin mRNA expression was shown to depend in part on activation of the EGFR/Akt signaling pathway [21]. However, it remains unclear whether *Lactobacillus* mediates this effect and the inhibition of ETEC infection via activation of EGFR and its downstream targets.

In this study, we hypothesized that probiotic *L*. *rhamnosus* ATCC 7469 regulates the inflammatory response of porcine intestinal epithelial J2 (IPEC-J2) cells and aids in maintaining the intestinal barrier through modulating TLR/NLR cooperation and EGFR/Akt signaling to protect IECs from the deleterious effects of ETEC infection. The aim of this study therefore was to investigate the effects of *Lactobacillus* administration on IEC physiology. Our goal is to provide a rationale for the use of probiotics as therapeutic and preventative agents, at least for infectious diarrhea, and perhaps also for other diseases associated with mucosal inflammation.

## Materials and Methods

### Cell line and culture conditions

The porcine intestinal epithelial J2 cell line (IPEC-J2, ACC701, DSMZ) was kindly supplied by Prof. Yanming Zhang of the Northwest A & F University in China. Cells were continuously maintained in culture. The IPEC-J2 cells used in this study represented non-transformed, polarized-growing porcine jejunal epithelial cells and were isolated from a neonatal, unsuckled piglet.

IPEC-J2 cells were cultured in Dulbecco’s Modified Eagle medium/Ham’s F-12 (1:1) medium supplemented with 10% heat-inactivated fetal calf serum (FCS) (Invitrogen, Carlsbad, CA) at 37°C in an atmosphere of 5% CO_2_ and 95% air at 95% relative humidity. For bacteria-free assays, an antibiotic mixture (100 U/ml penicillin and 100 μg/ml streptomycin; Invitrogen) was added to the culture medium. Undifferentiated cells reached confluence after 1–2 days. The IPEC-J2 cells were subcultured with PBS containing 0.25% trypsin and 0.5 mM EDTA (Invitrogen). For assays described below, IPEC-J2 cells were grown on transwell filters and cultured for 10 d after reaching confluence in medium without FCS to allow for differentiation. Under these culture conditions, the IPEC-J2 cells differentiated and exhibited enterocytic features, including microvilli and TJs, when grown on transwell filters. Medium was changed 3 times per week.

### Bacterial strains

*Lactobacillus rhamnosus* ATCC 7469 was purchased from the Chinese General Microorganism Culture Collection and grown in De Man, Rogosa, and Sharpe (MRS) broth (Oxoid, Hampshire, UK) for 24 h at 37°C under microaerophilic conditions. After overnight incubation, bacteria were diluted 1:100 in fresh MRS broth and grown for about 8 h until reaching mid-log phase, for all experiments.

The *Escherichia coli* F4-expressing strain (serotype O149:K91, K88ac) was obtained from the China Veterinary Culture Collection Center and grown in Luria-Bertani (LB) broth (Oxoid, Basingstoke, England). After overnight incubation at 37°C with vigorous shaking, bacteria were diluted 1:100 in fresh LB and grown for about 3 h until reaching mid-log phase.

### Bacterial adhesion assay

IPEC-J2 cells (5 × 10^5^ cells per well) were seeded onto a 6-well transwell collagen-coated PTFE filter (pore size 0.4 μm; 4.7 cm^2^; Corning, Corning City, NY). At day 10, confluent monolayers of cells cultured in medium supplemented with porcine mucin (0.5 mg/ml; Sigma-Aldrich, Saint Louis, MO) and without were treated under one of three conditions, as follows: (i) F4^+^ ETEC (10^7^ colony forming units [CFU]/ml) infection alone; (ii) simultaneous incubation with 1 ml of medium containing *L*. *rhamnosus* (10^8^ CFU/ml) and F4^+^ ETEC (10^7^ CFU/ml) infection; and (iii) pre-incubation with 1 ml of medium containing *L*. *rhamnosus* (10^8^ CFU/ml) for 2 h prior to addition of F4^+^ ETEC (10^7^ CFU/ml). We chose the bacterial concentration and time of incubation based on preliminary experiments to allow for bacterial adhesion and membrane damage without disruption of the cell monolayers. At 3 h following F4^+^ ETEC (10^7^ CFU/ml) challenge, the number of F4^+^ ETEC CFU recovered was determined. After incubation, cells were washed with PBS, lysed, and homogenized with 0.1% (v/v) Triton X-100 (Sigma-Aldrich) in ddH_2_O and plated on LB agar after serial dilution. Plates were incubated overnight at 37°C, after which the number of CFU was determined. Preliminary experiments confirmed that *L*. *rhamnosus* did not form colonies after overnight incubation on LB agar at 37°C under aerobic conditions.

To assay competitive adhesion to mucin, 300 μl of porcine mucin (0.5 mg/ml) in sterile PBS was immobilized passively on Maxisorp microtiter plate wells (Nunc, Roskilde, Denmark) by overnight incubation at 4°C. Wells were then washed twice with PBS to remove unbound mucin. The densities of *L*. *rhamnosus* (10^8^ CFU/ml) and F4^+^ ETEC (10^7^ CFU/ml) were adjusted using sterile PBS. Next, 200 μl of a culture of each strain was added to wells coated with mucin as described above and allowed to adhere for 3 h at 37°C. Non-adhering bacteria were then withdrawn, and the wells were washed five times with 300 μl of sterile PBS. Adhered F4^+^ ETEC were released using 300 μl of 0.1% (v/v) Triton X-100 and then enumerated on LB agar.

### Mucin production

IPEC-J2 cells (10^6^ cells per filter) differentiated on a 6-well transwell collagen-coated PTFE filter were treated under one of five conditions, as follows: (i) medium; (ii) F4^+^ ETEC (10^7^ CFU/ml) infection alone; (iii) *L*. *rhamnosus* (10^8^ CFU/ml) incubation alone; (iv) simultaneous incubation with 1 ml of medium containing *L*. *rhamnosus* (10^8^ CFU/ml) and F4^+^ ETEC (10^7^ CFU/ml) infection; and (v) pre-incubation with 1 ml of medium containing *L*. *rhamnosus* (10^8^ CFU/ml) for 2 h prior to addition of F4^+^ ETEC (10^7^ CFU/ml). At 3 h after F4^+^ ETEC challenge, IPEC-J2 cells were harvested and fixed with 4% paraformaldehyde at 4°C for 20 min. Acidic mucopolysaccharides were stained with Alcian Blue (AB) at pH 2.5, and neutral mucopolysaccharides were visualized using the periodic acid-Schiff (PAS) reaction (Luoji Biotech, Beijing, China), as described by the manufacturer. AB stains acidic glycoproteins blue and PAS stains neutral glycoproteins pink, whereas mixtures of neutral and acidic mucin glycoproteins appear purple. For semi-quantitative determination of mucin (purple) production, digital images were analyzed using Image Pro Plus 6.0 software (Media Cybernetics, Rockville, MD), allowing quantification of mucin levels as the mean integrated optical density (IOD). Results are presented as the ratio of purple mucin IOD to blue mucin IOD.

### Apoptosis assay

Apoptosis of IPEC-J2 cells was assessed using an apoptosis kit with fluorescein isothiocyanate (FITC)-conjugated annexin V and propidium iodide (PI) for flow cytometry (Invitrogen). At 3 h after F4^+^ ETEC challenge, IPEC-J2 cells were harvested, washed in pre-chilled PBS, and stained with FITC-conjugated annexin V (5 μl) and PI (1 μl) in succession for 10 min at room temperature. Appropriate single-labeled and unlabeled controls were used. After filtering through a 70-μm nylon cell strainer (BD Biosciences, San Jose, CA), cells were assessed for fluorescence using a FACScalibur flow cytometer (BD Biosciences) equipped with FlowJo software. The percentages of early apoptotic (annexin-FITC-positive/PI-negative) and late apoptotic (annexin-FITC/PI-double positive) cells were determined.

### Western blotting

At 3 h after F4^+^ ETEC challenge, IPEC-J2 cells were lysed in 0.5 ml of cold RIPA buffer (150 mM sodium chloride, 1.0% Igepal CA-630, 0.5% sodium deoxycholate, 0.1% sodium dodecyl sulfate, 50 mM Tris-HCl, pH 8.0) supplemented with complete protease inhibitors (104 mM AEBSF, 80 μM aprotinin, 4 mM bestatin, 1.4 mM E-64, 2 mM leupeptin, and 1.5 mM pepstatin A) (Sigma-Aldrich). The scraped cell suspensions were centrifuged at 10,000 × *g* for 15 min at 4°C to remove insoluble debris, and the supernatant was used for Western blot analysis. Protein concentrations were determined using the Bio-Rad DC protein assay kit II (Bio-Rad Laboratories, Hercules, CA). The primary antibodies were rabbit anti-ZO-1 (ab59720), rabbit anti-occludin (ab31721), mouse anti-heat shock protein 72 ([Hsp72], ab2787; Abcam, Cambridge, UK), anti-phospho-Ser473 (p)-Akt (ab138726), rabbit anti-phospho-Tyr124 (p)-PKCα (ab32376), rabbit anti-phospho-Tyr1068 (p)-EGFR (ab32430; Epitomics, Burlingame, CA), rabbit anti-total-EGFR (18986-1-AP), anti-total-Akt (10176-2-AP), and mouse anti- glyceraldehyde-3-phosphate dehydrogenase ([GAPDH], 60004-1-Ig) (Proteintech Group, Chicago, IL). Horseradish peroxidase (HRP)-conjugated affinipure goat anti-mouse IgG (H+L) (SA00001-1) or goat anti-rabbit IgG (H+L) (SA00001-2; Proteintech Group) were used as secondary antibodies. GAPDH served as an internal control and exhibited stable expression regardless of treatment. The optical density (OD) of each band was quantified by densitometric analysis using Quantity One software (Bio-Rad Laboratories). Results are presented as the ratio of the p-EGFR or p-Akt band intensity to the total EGFR or total Akt band intensity, respectively, and the ratio of the p-PKCα, ZO-1, and occludin band intensity to the GAPDH band intensity.

### Quantitative real-time PCR

IPEC-J2 cells were collected at 3 h after F4^+^ ETEC challenge. Total RNA was extracted from the cells using Trizol reagent (Invitrogen). The integrity of extracted RNA was confirmed on agarose gel electrophoresis by staining with ethidium bromide and visualization under UV light. The quantity and purity (OD_260_/OD_280_ absorption ratio >1.9) of RNA was determined using a NanoDrop ND-2000C spectrophotometer (NanoDrop Technologies Inc., Wilmington, DE). A 2-μg aliquot of total RNA was reverse-transcribed into cDNA with 200 U of M-MLV using 1 μg of oligo (dT)_15_ primer, 10 mM dNTP mix, M-MLV 5× reaction buffer, and 25 U of rRNasin ribonuclease inhibitor (Promega, Madison, WI) in a final volume of 25 μL. To detect DNA contamination, a negative control (without enzyme) was included. Synthesized cDNA was stored at −20°C prior to real-time PCR analysis.

Quantitative real-time PCR was performed using an ABI 7500 real-time PCR system (Applied Biosystems, Foster City, CA). Primer sequences are listed in Table 1. The cDNA was amplified in triplicate with SYBR Premix DimerEraser (TakaRa Biotechnology Inc., Shiga, Japan). A non-template control of nuclease-free water was included in each run.

**Table 1:** Sequences of oligonucleotide primers used for real-time PCR, length of the respective PCR product, and gene accession number.

Gene product^a^	Primer		Product size (bp)	Accession number
	Direction^b^	Sequence (5' to 3')		
GADPH	F	CCAGAACATCATCCCTGCTT	229	NM_001206359
	R	GTCCTCAGTGTAGCCCAGGA		
TLR2	F	TCACTTGTCTAACTTATCATCCTCTTG	162	XM_005653579
	R	TCAGCGAAGGTGTCATTATTGC		
TLR4	F	GCCATCGCTGCTAACATCATC	108	NM_001113039
	R	CTCATACTCAAAGATACACCATCGG		
TLR9	F	GTGGAACTGTTTTGGCATC	199	NM_213958
	R	CACAGCACTCTGAGCTTTGT		
NOD1	F	ACCGATCCAGTGAGCAGATA	140	NM_001114277
	R	AAGTCCACCAGCTCCATGAT		
NOD2	F	GAGCGCATCCTCTTAACTTTCG	66	NM_001105295
	R	ACGCTCGTGATCCGTGAAC		

^a ^GADPH = glyceraldehyde-3-phosphate dehydrogenase; TLR = toll-like receptor; NOD = nucleotide-binding oligomerization domain.

^b^ F = forward; R = reverse.

Relative quantification of mRNA expression was assessed by normalizing the cycle threshold (C_T_) values of the target genes to the C_T_ values of the housekeeping gene encoding GAPDH. The results are presented as fold change using the 2^−ΔΔCT^ method. The relative mRNA expression of target genes in each group was calculated using the following equations: ΔC_T_ = C_T (target gene)_ — C_T (GAPDH)_, or ΔΔC_T_ = ΔC_T (treated group)_ — ΔC_T (control group)_.

### ELISA

The concentrations of IL-10, TNF-α, and PGE_2_ in supernatants from IPEC-J2 cell cultures at 3 h after F4^+^ ETEC challenge were determined using commercially available ELISA kits specific for porcine TNF-α, porcine IL-10 (R&D Systems, Minneapolis, MN), and porcine PGE_2_ (Cayman Chemical Co., Ann Arbor, MI).

### Statistical analysis

Statistical evaluations were performed using the SAS statistical software package, version 9.1 (SAS Institute Inc., Cary, NC). Data were also evaluated using ANOVA procedures. With regard to small sample sizes, normal distribution and homogeneity of variance were assumed with the UNIVARIATE (Shapiro-Wilk test) and HOVTEST procedures. Natural logarithm transformation was performed prior to analysis for IL-10 and PGE_2_ ELISA data to yield a normal distribution. Differences between means were compared using Tukey’s honestly significant difference (HSD) post hoc test. Data were visualized using GraphPad Prism 5 software (Graphpad Software Inc., San Diego, CA). A *P*-value of <0.05 was considered indicative of statistical significance. All experiments were performed three times.

## Results

### Pre-incubation with *L*. *rhamnosus* reduced the adhesion of F4^+^ ETEC

**Fig 1:**
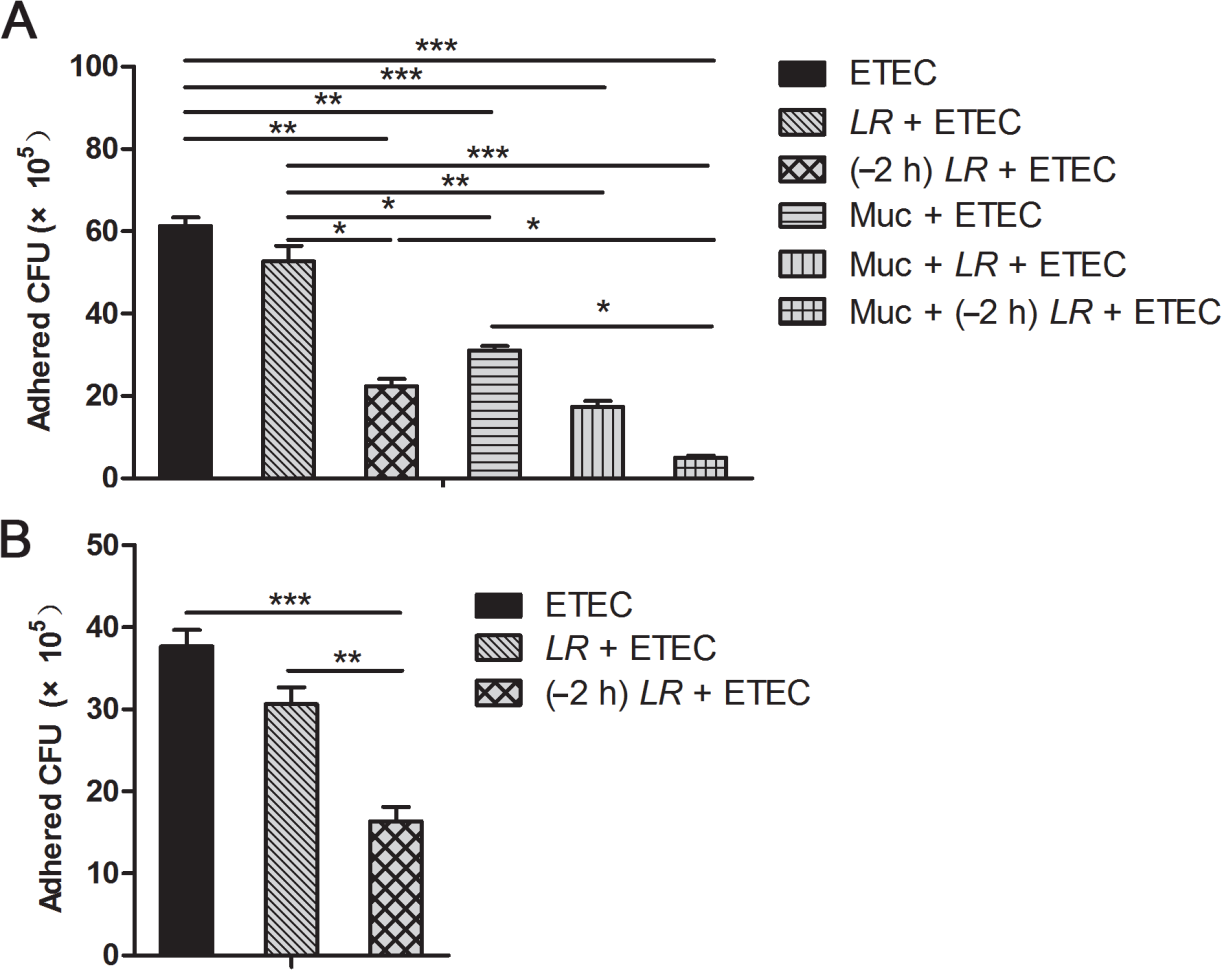
Incubation with *L*. *rhamnosus* reduces adhesion of F4^+^ ETEC to the IPEC-J2 cell monolayer. IPEC-J2 cells cultured in medium with and without porcine mucin were subjected F4^**+**^ ETEC challenge alone (ETEC), co-incubated with *L*. *rhamnosus* plus F4^**+**^ ETEC challenge simultaneously (*LR* + ETEC), or pre-incubated with *L*. *rhamnosus* for 2 h followed by F4^**+**^ ETEC challenge [(–2 h) *LR* + ETEC]. At 3 h following F4^**+**^ ETEC (10^**7**^ CFU/ml) challenge, the number of F4^**+**^ ETEC CFUs recovered from IPEC-J2 cells was determined (A) and the number of F4^**+**^ ETEC CFUs recovered from mucin-coated microtiter plate wells with and without *L*. *rhamnosus* incubation was determined (B). Data are presented as means ± SEM of three independent experiments. *P < 0.05, **P < 0.01, ***P < 0.001.

To investigate the effect of *L*. *rhamnosus* on adhesion of F4^+^ ETEC to IPEC-J2 cells and *L*. *rhamnosus* competition with mucin, we examined the adhesion of F4^+^ ETEC to IPEC-J2 cells in the absence or presence of mucin coated in microtiter plate wells (Fig 1A and 1B).

In both the absence and presence of mucin, fewer F4^+^ ETEC CFU were recovered from IPEC-J2 cells pre-incubated with *L*. *rhamnosus* as compared with IPEC-J2 cells only infected with F4^+^ ETEC (*P* = 0.003 and *P* = 0.034, respectively, Fig 1A). The number of F4^+^ ETEC CFU recovered from IPEC-J2 cells pre-incubated with *L*. *rhamnosus* was lower compared with IPEC-J2 cells co-incubated with *L*. *rhamnosus* in the absence of mucin (*P* = 0.031). Mucin addition resulted in a decrease in the number of F4^+^ ETEC CFU recovered from IPEC-J2 cells infected with F4^+^ ETEC, co-incubated with *L*. *rhamnosus*, and pre-incubated with *L*. *rhamnosus* (*P* = 0.008, *P* = 0.006, and *P* = 0.039, respectively). Furthermore, fewer CFU were recovered from IPEC-J2 cells co- or pre-incubated with *L*. *rhamnosus* in the presence of mucin than from cells only infected with F4^+^ ETEC (*P* < 0.001). Also, fewer CFU were recovered from IPEC-J2 cells infected with F4^+^ ETEC or pre-incubated with *L*. *rhamnosus* in the presence of porcine mucin than from cells co-incubated with *L*. *rhamnosus* in the absence of mucin (*P* = 0.043 and *P* < 0.001, respectively).

In the assay of competitive adhesion to mucin, pre-incubation with *L*. *rhamnosus* resulted in a decrease in the number of CFU recovered compared with F4^+^ ETEC challenge alone or co-incubation with *L*. *rhamnosus* (*P* < 0.001 and *P* = 0.005, respectively; Fig 1B).

### Effect of *L*. *rhamnosus* on the mucin layer

**Fig 2:**
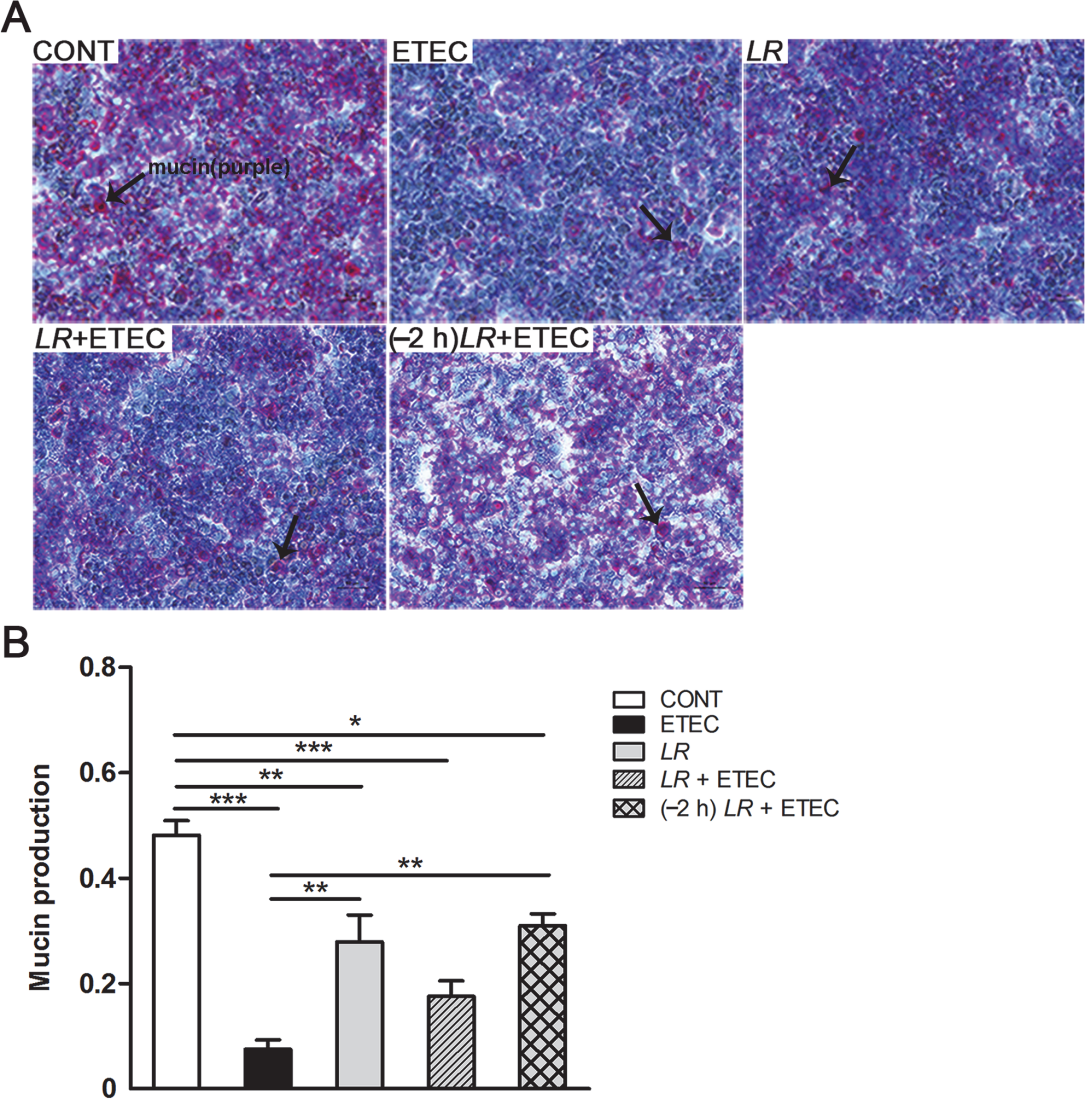
Effect of *L*. *rhamnosus* on the mucin layer. (A) IPEC-J2 cells were cultured with medium alone (CONT), F4^**+**^ ETEC (ETEC), *L*. *rhamnosus* (*LR*), *L*. *rhamnosus* plus F4^**+**^ ETEC simultaneously (*LR* + ETEC), or pre-incubated with *L*. *rhamnosus* for 2 h followed by F4^**+**^ ETEC challenge [(–2 h) *LR* + ETEC]. Mucin production (purple clusters, arrows) was determined by AB-PAS staining of the indicated cultures at 3 h after F4^**+**^ ETEC challenge. (B) Semi-quantitative determination of mucin (purple) production. Digital images were analyzed using Image Pro Plus 6.0 software, which enabled quantification of mucin level as the mean of integrated optical density (IOD). The ratio of purple mucin IOD to blue mucin IOD was calculated. Data are presented as means ± SEM of three independent experiments. *P < 0.05, **P < 0.01, ***P < 0.001. Scale bars, 100 μm.

Mucin glycoproteins are the main components of the first barrier that bacteria encounter in the intestinal tract. The effect of *L*. *rhamnosus* on the mucin layer in IPEC-J2 cells with and without F4^+^ ETEC infection was evaluated using AB-PAS staining (Fig 2). Untreated IPEC-J2 control cells were lined by a continuous, homogeneous, purple mucin layer (Fig 2A). After exposure to F4^+^ ETEC, the continuous purple mucin layer became disrupted, and mucin production was reduced in IPEC-J2 cells infected with F4^+^ ETEC alone and in cells co- or pre-incubated with *L*. *rhamnosus*, compared with untreated IPEC-J2 control cells (*P* < 0.001, *P* < 0.001, and *P* = 0.013, respectively; Fig 2A and 2B). Pre-incubation with *L*. *rhamnosus* attenuated F4^+^ ETEC-induced mucin layer disruption (Fig 2A). Although IPEC-J2 cells incubated with *L*. *rhamnosus* only exhibited lowered mucin production compared with untreated IPEC-J2 control cells (*P* = 0.004), mucin production was higher in cells incubated with *L*. *rhamnosus* only and in cells pre-incubated with *L*. *rhamnosus* than in cells only infected with F4^+^ ETEC (*P* = 0.006 and *P* = 0.003, respectively; Fig 2B).

### Effect of *L*. *rhamnosus* on apoptosis of IPEC-J2 cells

**Fig 3:**
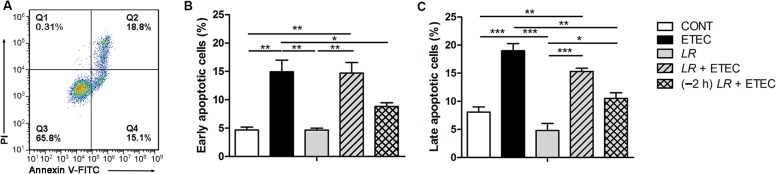
Effect of *L*. *rhamnosus* on apoptosis of IPEC-J2 cells. IPEC-J2 cells were collected from the indicated cultures at 3 h after F4^**+**^ ETEC challenge. Apoptosis was assessed by flow cytometry. (A) Representative two-dimensional scatter plots of annexin V versus propidium iodide. (B) The percentage of early apoptotic cells. (C) The percentage of late apoptotic cells. Data are presented as means ± SEM of three independent experiments. *P < 0.05, **P < 0.01, ***P < 0.001.

Intestinal epithelial cells commonly undergo apoptosis in response to injurious stimuli (microbial, hypoxic, or chemical), allowing for the dismantling of damaged cells without the release of cellular contents and activation of the immune system. Excessive or inappropriate apoptosis may lead to damage of the intestinal barrier, however. Cells exposed to F4^+^ ETEC alone had a higher percentage of early and late apoptosis compared with untreated IPEC-J2 controls (*P* = 0.002 and *P* < 0.001, respectively; Fig 3). Pre-incubation (but not co-incubation) with *L*. *rhamnosus* resulted in a decrease in the percentage of early and late apoptosis during F4^+^ ETEC infection (*P* = 0.048 and *P* = 0.001, respectively). The percentages of early and late apoptosis were higher in cells co-incubated with *L*. *rhamnosus* than in untreated IPEC-J2 controls (*P* = 0.002 and *P* = 0.002, respectively) or cells incubated with *L*. *rhamnosus* alone (*P* = 0.004 and *P* < 0.001, respectively).

### Effect of *L*. *rhamnosus* on *TLR* and *NOD* mRNA expression

**Fig 4:**
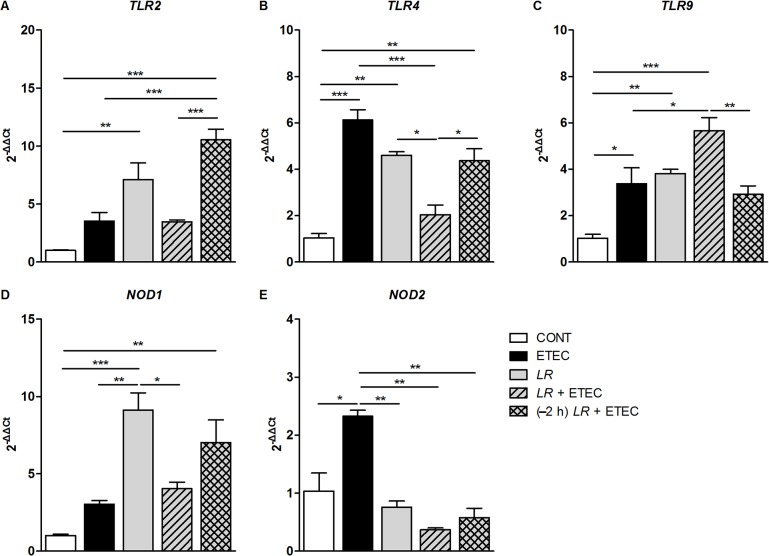
Treatment with *L*. *rhamnosus* alters *TLR* and *NLR* expression after F4^+^ ETEC infection. IPEC-J2 cells were collected from the indicated cultures at 3 h after F4^**+**^ ETEC challenge. The relative expression of mRNAs for genes encoding (A) *TLR2*, (B) *TLR4*, (C) *TLR9*, (D) *NOD1*, and (E) *NOD2* was analyzed by quantitative real-time PCR. Data are presented as means ± SEM of three independent experiments. *P < 0.05, **P < 0.01, ***P < 0.001.

To understand the interaction between IPEC-J2 inflammatory responses and the effect of *L*. *rhamnosus* in preventing F4^+^ ETEC infection, we quantified the relative expression of mRNAs for selected genes encoding *TLRs* and *NODs* (Fig 4).

Incubation with *L*. *rhamnosus* alone increased relative expression of *TLR2* and *TLR4* mRNAs (*P* = 0.002 and *P* = 0.003, respectively; Fig 4A and 4B). The relative expression of *TLR2* mRNA was significantly higher in cells pre-incubated with *L*. *rhamnosus* during F4^+^ ETEC infection than in either untreated IPEC-J2 controls or cells only infected with F4^+^ ETEC (*P* < 0.001). The relative expression of *TLR2* mRNA in cells pre-incubated with *L*. *rhamnosus* was significantly higher than in cells co-incubated with *L*. *rhamnosus* during F4^+^ ETEC infection (*P* < 0.001).

As expected, an F4^+^ ETEC-induced increase (*P* < 0.001) in the relative expression of *TLR4* mRNA was observed, and this increase was attenuated (*P* < 0.001) by co-incubation with *L*. *rhamnosus*. The relative expression of *TLR4* mRNA was higher in cells pre-incubated with *L*. *rhamnosus* than in untreated IPEC-J2 controls or in cells co-incubated with *L*. *rhamnosus* (*P* = 0.002 and *P* = 0.031, respectively).

The relative expression of *TLR9* mRNA was elevated in cells treated with F4^+^ ETEC alone, cells incubated with *L*. *rhamnosus* alone, and cells co-incubated with F4^+^ ETEC and *L*. *rhamnosus* compared with untreated IPEC-J2 controls, but *TLR9* mRNA expression was not elevated in cells pre-incubated with *L*. *rhamnosus* (*P* = 0.025, *P* = 0.009, and *P* < 0.001, respectively; Fig 4C). The relative expression of *TLR9* mRNA was higher in cells co-incubated with *L*. *rhamnosus* than in cells infected with F4^+^ ETEC and cells pre-incubated with *L*. *rhamnosus* (*P* = 0.031 and *P* = 0.013, respectively).

Cells incubated with *L*. *rhamnosus* alone and those pre-incubated with *L*. *rhamnosus* exhibited higher relative expression of *NOD1* mRNA than did untreated IPEC-J2 controls (*P* < 0.001 and *P* = 0.004; Fig 4D), but cells infected with F4^+^ ETEC alone did not. The relative expression of *NOD1* mRNA was higher in cells incubated with *L*. *rhamnosus* alone than in cells infected with F4^+^ ETEC and co-incubated with *L*. *rhamnosus* (*P* = 0.002 and *P* = 0.038, respectively). F4^+^ ETEC infection led to an increase in the relative expression of *NOD2* mRNA, but *L*. *rhamnosus* incubation did not (*P* = 0.043; Fig 4E). The F4^+^ ETEC-induced increase in *NOD2* mRNA expression was attenuated by co- or pre-incubation with *L*. *rhamnosus* (*P* = 0.002 and *P* = 0.003, respectively).

### Concentrations of TNF-α, IL-10, and PGE_2_ in IPEC-J2 cell culture supernatants

**Fig 5:**
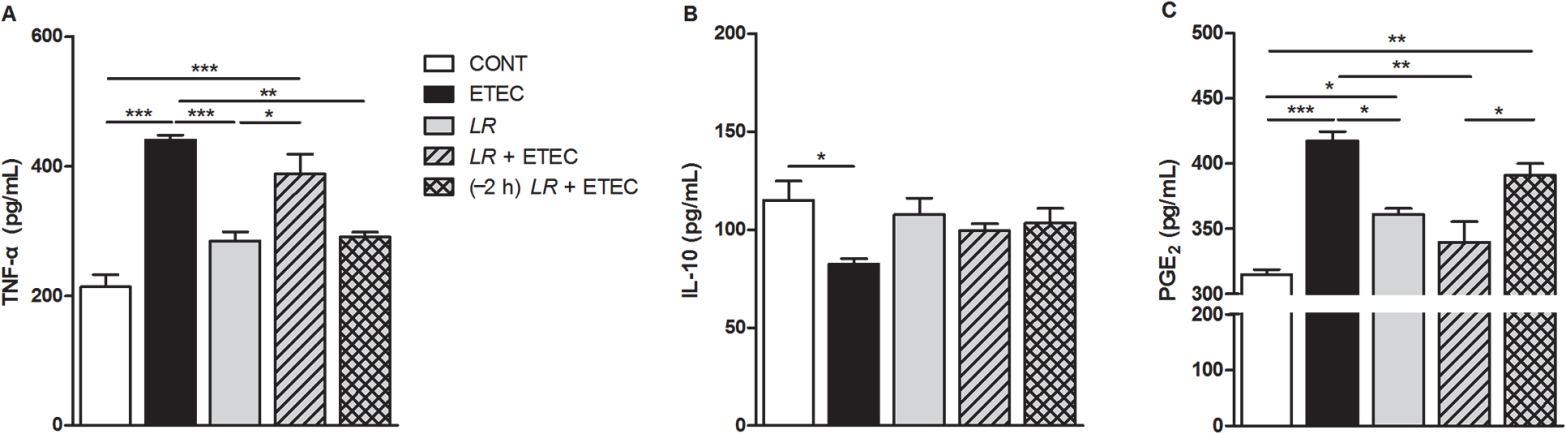
Concentrations of TNF-α, IL-10, and PGE_2_ in IPEC-J2 cell culture supernatants. Supernatants were collected from the indicated cultures at 3 h after F4^**+**^ ETEC challenge. The concentrations of (A) TNF-α, (B) IL-10, and (C) PGE_**2**_ were determined by ELISA. Data are presented as means ± SEM of three independent experiments. *P < 0.05, **P < 0.01, ***P < 0.001.

To test whether *L*. *rhamnosus* can attenuate the inflammatory response induced by F4^+^ ETEC, we measured the concentrations of TNF-α, IL-10, and PGE_2_ in the supernatant from cultures of treated IPEC-J2 cells and untreated controls (Fig 5). TNF-α production was increased both in cells infected with F4^+^ ETEC alone and in cells co-incubated with F4^+^ ETEC and *L*. *rhamnosus* compared with untreated IPEC-J2 controls (*P* < 0.001; Fig 5A). F4^+^ ETEC infection resulted in a decrease in the IL-10 supernatant concentration (*P* = 0.043), but pre-treatment with *L*. *rhamnosus* prevented this decrease (Fig 5B). Compared with untreated IPEC-J2 controls, F4^+^ ETEC-infected cells or F4^+^ ETEC-infected cells pre-incubated with *L*. *rhamnosus* secreted a significantly higher amount of PGE_2_ (*P* < 0.001 and *P* = 0.001, respectively; Fig 5C). However, although the PGE_2_ concentration in the supernatant of cells co-incubated with *L*. *rhamnosus* was elevated, the difference was not significant.

### Effect of *L*. *rhamnosus* on p-EGFR, p-Akt, and p-PKCα protein expression

**Fig 6:**
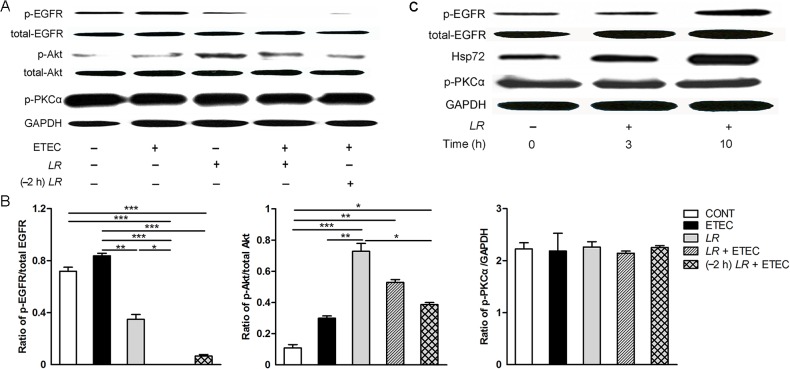
Western blot detection of phosphorylation of EGFR, Akt, PKCα, and Hsp72. (A) Representative panels of p-EGFR, total-EGFR, p-Akt, total-Akt, and p-PKCα proteins in IPEC-J2 cells collected from the indicated cultures at 3 h after F4^**+**^ ETEC challenge. (B) The intensities of p-EGFR, total-EGFR, p-Akt, total-Akt, and p-PKCα bands were determined using Quantity One software. Results are presented as the ratio of the p-EGFR band intensity to the total-EGFR band intensity, the ratio of the p-Akt band intensity to the total-Akt band intensity, and the ratio of p-PKCα band intensity to the GAPDH band intensity. (C) Representative panels of heat shock protein 72 (Hsp72), p-EGFR, and p-PKCα in IPEC-J2 cells treated with *L*. *rhamnosus* alone for 0, 3, and 10 h. Expression of GAPDH was measured as an internal control. Data are presented as means ± SEM of three independent experiments. *P < 0.05, **P < 0.01, ***P < 0.001.

EGFR affects the survival of epithelial cells through multiple signaling pathways. We therefore examined the effect of *L*. *rhamnosus* on activation of EGFR and the downstream regulatory pathway molecules Akt and PKCα (Fig 6).

Expression of p-EGFR was decreased in cells co- or pre-incubated with *L*. *rhamnosus* during F4^+^ ETEC infection compared with untreated IPEC-J2 controls as well as cells infected with F4^+^ ETEC alone (*P* < 0.001). IPEC-J2 cells only incubated with *L*. *rhamnosus* also exhibited lower p-EGFR protein expression compared with cells only infected with F4^+^ ETEC (*P* = 0.005). In contrast, p-Akt expression was elevated in cells incubated with *L*. *rhamnosus* only and cells co- or pre-incubated with *L*. *rhamnosus*, compared with untreated IPEC-J2 controls (*P* < 0.001, *P* = 0.008, and *P* = 0.022, respectively; Fig 6A and 6B). Expression of p-Akt was higher in cells incubated with *L*. *rhamnosus* only than in cells only infected with F4^+^ ETEC (*P* = 0.006). No differences in p-PKCα expression were observed among the different groups.

Increased p-EGFR and Hsp72 protein expression was observed at 10 h only in IPEC-J2 cells incubated with *L*. *rhamnosus* alone (Fig 6C).

### Effect of *L*. *rhamnosus* on ZO-1 and occludin protein expression

**Fig 7:**
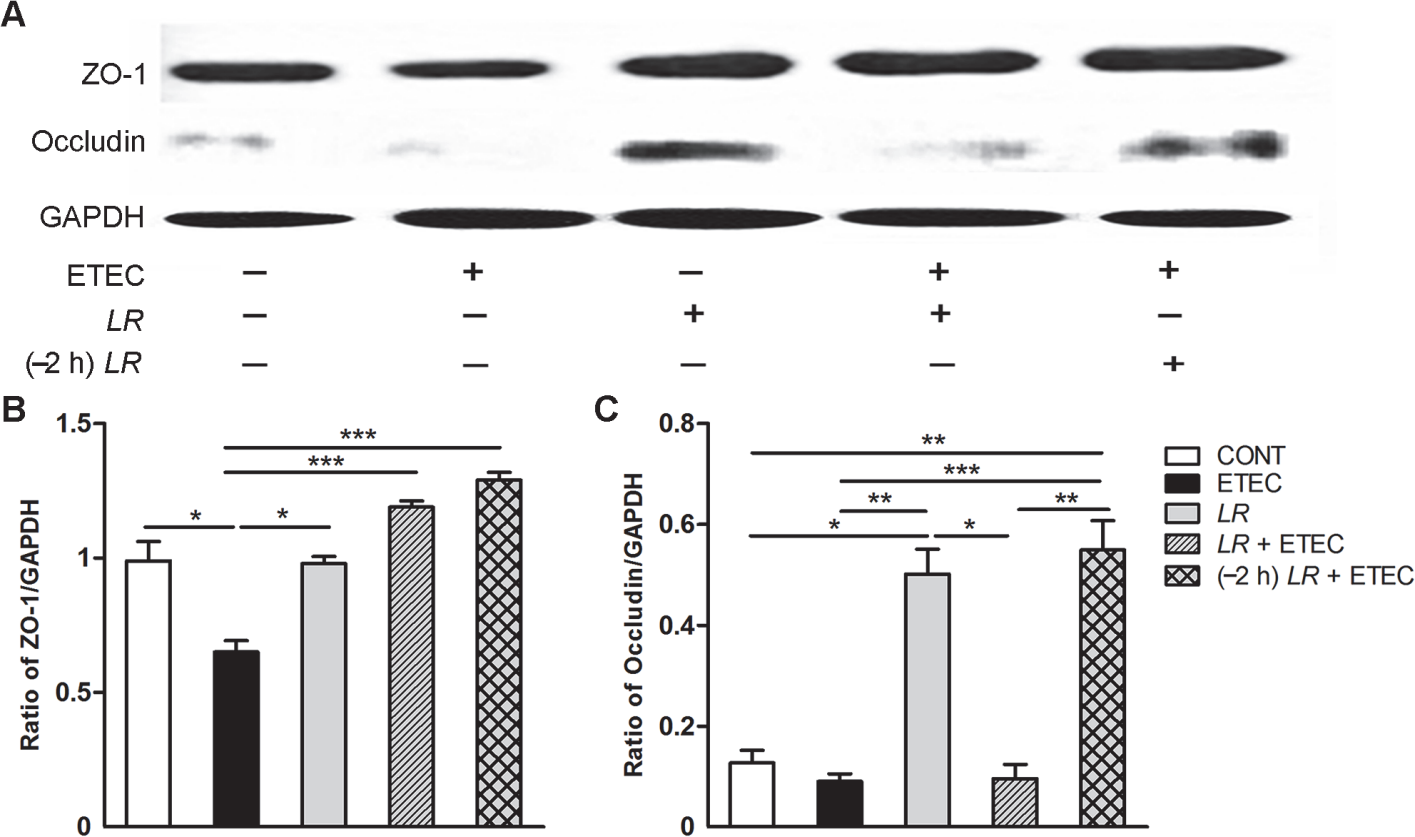
Western blot detection of tight junction proteins. (A) Representative panels of zonula occludens-1 (ZO-1) and occludin proteins in IPEC-J2 cells collected from the indicated cultures at 3 h after F4^**+**^ ETEC challenge. Expression of GAPDH was measured as an internal control. Results are presented as the ratio of the (B) ZO-1 band intensity and (C) occludin band intensity to the GAPDH band intensity. Data are presented as means ± SEM of three independent experiments. *P < 0.05, **P < 0.01, ***P < 0.001.

The TJ plays a fundamental role in membrane barrier function and integrity through interaction of the ZO protein with the transmembrane protein occludin and the apical perijunctional actomyosin ring. To investigate the effect of *L*. *rhamnosus* on TJ integrity, we examined ZO-1 and occludin protein expression in IPEC-J2 cells with and without pre- or co-incubation with *L*. *rhamnosus* during F4^+^ ETEC infection (Fig 7).

F4^+^ ETEC infection resulted in a decrease in ZO-1 protein expression (*P* = 0.022), and this decrease was attenuated by co- or pre-incubation with *L*. *rhamnosus* (*P* < 0.001; Fig 7A and 7B). ZO-1 protein expression was higher in cells only incubated with *L*. *rhamnosus* than in cells only infected with F4^+^ ETEC (*P* = 0.031).

Occludin protein expression was elevated in cells only incubated with *L*. *rhamnosus* and cells pre-incubated with *L*. *rhamnosus* compared with untreated IPEC-J2 controls (*P* = 0.018 and *P* = 0.009, respectively; Fig 7A and 7C). Compared with cells only infected with F4^+^ ETEC and cells co-incubated with *L*. *rhamnosus*, occludin protein expression was higher in cells only incubated with *L*. *rhamnosus* (*P* = 0.004 and *P* = 0.012, respectively) and cells pre-incubated with *L*. *rhamnosus* (*P* < 0.001 and *P* = 0.007, respectively).

## Discussion

Certain probiotics have the potential to serve as alternatives to antibiotics for preventing enteric infections. However, the mechanism underlying the benefits derived from biotherapy with *Lactobacillus* is incompletely understood. In this study, we explored the effects of *L*. *rhamnosus* on the epithelial barrier and inflammatory response in IPEC-J2 cells. *Lactobacillus rhamnosus* inhibited F4^+^ ETEC adhesion and ameliorated F4^+^ ETEC-induced mucin layer destruction in these cells. Moreover, *L*. *rhamnosus* attenuated F4^+^ ETEC-induced damage to the IPEC-J2 cell barrier by suppressing F4^+^ ETEC-induced apoptosis and increasing ZO-1 and occludin protein expression. These data indicated that the dosage of *L*. *rhamnosus* used in this study was within the effective range for IPEC-J2 cells.

In agreement with a previous study [22], the present study showed that pre-treatment of IPEC-J2 cells with *L*. *rhamnosus* inhibits the adhesion of F4^+^ ETEC and that addition of exogenous mucin enhances the capacity of *L*. *rhamnosus* to reduce the adhesion of F4^+^ ETEC to IPEC-J2 cells. The mucus layer is the first barrier pathogens encounter upon contact with epithelial cells. Elaboration of mucus within minutes or hours of insult is considered a major part of IEC innate defenses. Mucin proteins are believed to protect epithelial cells from microbial pathogens by limiting their access to the cells through simple steric hindrance, by providing a physicochemical barrier, or through specific mucin-bacterial interactions [23]. Increased secretion of MUC3 mucin leads to reduced adhesion of enteropathogenic *Escherichia coli* (EPEC) and enterohemorrhagic *Escherichia coli* (EHEC) strains [24,25]. It has been shown that Spac pilin, located at the pilus, is essential for mucus interaction with LGG [26]. A recent study also showed that a C-terminal LPxTG motif–containing protein accounts for the binding of *L*. *reuteri* ATCC PTA 6475 to Caco-2 cells and mucus [27]. Moreover, the bacterial exopolysaccharide reuteran, synthesized by *Lactobacillus reuteri*, reduces ETEC colonization of piglet jejunal epithelial cells [28]. These data suggest that competition for attachment sites on mucus and amelioration of mucin layer destruction via *L*. *rhamnosus* play vital roles in preventing F4^+^ ETEC adhesion.

We found that pre-incubation, rather than co-incubation, with *L*. *rhamnosus* reduced F4^+^ ETEC adhesion. Indeed, a previous study found that pre-treatment with probiotics (*Lactobacillus acidophilus* and *Streptococcus thermophilus*) markedly reduced enteroinvasive *Escherichia coli* (EIEC) attachment and spread, whereas simultaneous addition of probiotics with EIEC had a lesser effect, and addition of probiotics 1 hour after EIEC challenge failed to reduce invasion [29]. Probiotics also differ with respect to production of autoinducers that can condition the formation of complex biofilms above the epithelial surface, conceivably modulating mucosal responses to the lumenal environment [30]. Production of the HSP GroEL in the biofilm was shown to enhance the immunomodulatory effects of *L*. *casei* ATCC334 [31]. These findings emphasize the importance of investigating a diversity of host-microorganism interactions when profiling probiotics.

In our study, *L*. *rhamnosus* pre-treatment suppressed F4^+^ ETEC-induced apoptosis of IECs, suggesting that *L*. *rhamnosus* prevents F4^+^ ETEC-induced apoptosis. Basal epithelial cell extrusion in response to apoptotic stimuli is a normal physiological event in the gastrointestinal tract and does not affect intestinal epithelial barrier integrity; however, an increased epithelial apoptosis rate might compromise the epithelial barrier.

A recent study showed that canonical TLR2 signaling may offer a novel therapeutic approach to barrier recovery in atopic dermatitis and ulcerative colitis [32,33]. In a *Citrobacter rodentium*–induced colitis model, TLR2-deficient mice exhibited 45 to 75% mortality coincident with severe defects in TJ-associated IEC integrity [34]. *Lactobacillus plantarum* enhances TJ function by rearrangement of TJ protein conformation in response to TLR2 signaling [35]. We found that pre-incubation with *L*. *rhamnosus* following F4^+^ ETEC infection or incubation with *L*. *rhamnosus* alone increased *TLR2* mRNA expression. Activation of TLR2 signaling triggered by *L*. *rhamnosus* is most likely mediated by lipoteichoic acid, lipoproteins, and peptidoglycans from gram-positive bacteria through formation of a heterodimer with either TLR1 or TLR6 [36]. Invasive *Streptococcus pneumoniae* and *Haemophilus influenzae* were shown to exploit TLR2/TLR4-mediated downregulation of TJ components to facilitate translocation across the epithelium at 24-h post-inoculation [37]. In the present study, *L*. *rhamnosus* pre-treatment attenuated F4^+^ ETEC-induced TNF-α elevation, indicating that development of a competent TJ may also serve to limit TLR2-mediated inflammation that might be harmful during later stages of F4^+^ ETEC infection. *TLR2* expression was enhanced in the early stage of F4^+^ ETEC infection in response to pathogen-induced inflammation in impaired IPEC-J2 cells, as was early ZO-1 and occludin protein expression, which limited interactions between microbial antigens and the mucosal defense system.

Tight juctions are major determinants of epithelial paracellular permeability, and altered TJ permeability contributes to pathogen entry and a net efflux of ions and water. ETEC infection increases transepithelial permeability in early weaned pigs [38]. Heat-stable toxin b from ETEC has been shown to impair intestinal epithelial barrier function by causing the redistribution and/or fragmentation of ZO-1 and occludin [39]. The present data showed that ETEC infection leads to decreased expression of ZO-1 and occludin proteins in IPEC-J2 cells. Furthermore, *L*. *rhamnosus* enhanced the intestinal barrier related to the increased expression of ZO-1 and an increase in the abundance of occludin protein. We speculate that altered expression of TJ proteins and increased epithelial apoptosis contributed to epithelial barrier dysfunction, and certainly both mechanisms were blocked by probiotic therapy in this IPEC-J2 model.

Two different signaling cascade sequences originating from TLR2 signaling result in intestinal homeostasis and pathogen surveillance: NF-κB and PI3K activation, respectively. In this study, we found that *L*. *rhamnosus* enhanced Akt activation, thus activating the TLR2/Akt pathway in IECs. Binding of MyD88 adapter-like (Mal) protein to the p85 subunit of PI3K upon activation of the TLR2/TLR6 heterodimer leads to Akt phosphorylation. Activation of the PI3K/Akt pathway as a result of TLR2 activation has also been shown to augment epithelial barrier integrity. TLR2 promotes intestinal barrier function through redistribution of the TJ protein ZO-1 in response to stress-induced damage and suppresses apoptosis under control of the PI3K/Akt pathway [40].

Nod-like receptors are the cytoplasmic counterparts of TLRs, and both receptor types constitute a ‘*tour de force*’ of cellular defenses against encountered microbial motifs at the plasma membrane and within the cell [41]. In Caco-2 cells, NOD1 prevents IκB kinase and NF-κB activation in response to EIEC infection [42]. The present study showed that pre-incubation with *L*. *rhamnosus* induces *NOD1* mRNA expression, which is accompanied by up-regulation of *TLR2* and *TLR9* mRNA expression. Interestingly, incubation with *L*. *rhamnosus* inhibited the F4^+^ ETEC-induced increase in *NOD2* mRNA expression. NOD2 serves as a receptor for MDP, a small molecule derived from bacterial cell wall peptidoglycan [43]. Incubation with *L*. *rhamnosus* may inhibit internalization of MDP.

It has been reported that Mal contributes to maintenance of the intestinal epithelial barrier via activation of PKC and regulation of TJs mediated by TLR2, possibly involving translocation of the PKC isoforms PKCα and PKCδ to the TJ region [44,45]. In this study, *L*. *rhamnosus* had no effect on the expression of PKCα in IPEC-J2 cells. The PKC family consists of 11 isoforms, and these intracellular enzymes are located at or near the ZO complex. TJ assembly is regulated by a network of signaling pathways that may involve different PKC isoforms. A previous study showed that *L*. *rhamnosus* protection of TJs and the barrier function from hydrogen peroxide–induced insult is correlated with increased activation of PKCε and PKCβI [46]. In contrast, the probiotic *E*. *coli* strain Nissle 1917 was shown to prevent EPEC-induced decreases in ZO-2 expression and redistribution by silencing PKCζ activation [47]. Activation of distinct PKC isoforms may differentially affect cellular transport and the barrier function of the epithelium.

EGFR activation was shown to be associated with anti-apoptosis and cell survival [48,49]. In the present study, treatment with *L*. *rhamnosus* unexpectedly inhibited EGFR activation, which would not otherwise be adversely affected by F4^+^ ETEC at 3 h after infection. EGFR is predominantly localized along the basolateral sides of polarized IECs [50]. ETEC, however, is an extracellular pathogen that attaches to the apical side of epithelial cells. As shown in a previous study, EPEC-mediated activation of EGFR can be observed as early as 15 min post-infection in EPEC-treated non-polarized Caco-2 cells, whereas EGFR phosphorylation in polarized Caco-2 cells is not evident until 4 h after infection [49]. We found that activation of EGFR by *L*. *rhamnosus* was time dependent. ETEC-induced defective barrier function may be a pre-requisite for ETEC itself or its secreted proteins to access and activate the basolateral EGFR; *L*. *rhamnosus* counteracted ETEC-induced basolateral EGFR activation via maintaining the TJ barrier.

A previous study showed that activation of TLR4 by LPS induces Cox-2 expression and PGE_2_ production via MyD88 signaling, which in turn may stimulate epithelial proliferation via an EGFR-dependent mechanism [51]. *Lactobacillus acidophilus* increases Cox-2 expression and PGE_2_ secretion in intestinal cell lines [52]. Previous studies showed that *L*. *amylovorus* and *L*. *jensenii* suppress TLR4 inflammatory signaling triggered by ETEC through modulation of the negative regulators Tollip and the IL-1R–associated kinases M (IRAK-M), A20, Bcl-3, and MKP-1 [7,53]. Recently, an *in vitro* study using a mouse enterocyte model found that LPS-mediated TLR4 signaling could be inhibited by activation of TLR9 with bacterial DNA via the inhibitory kinase IRAK-M [54]. Dependent on interaction with four different E prostanoid (EP) receptor subtypes, PGE_2_ regulates many physiological functions of the gut, including mucosal protection, gastrointestinal secretion, and motility [55]. Constitutive PGE_2_ production via EP4 receptor signaling in the intestine appears to protect the integrity of the epithelial intestinal wall, presumably through enhancement of epithelial cell survival and regeneration of the epithelium. In addition, PGE_2_ elicits powerful immunosuppressive effects that contribute to the resolution of acute inflammation, facilitating tissue regeneration and the return to homeostasis.

It has been reported that the LGG-derived protein p40 up-regulates *Muc2* gene expression and increases mucus production through transactivation of EGFR/Akt signaling in LS174T human colon cancer cells [56]. LGG-derived p40 protein stimulates release of the EGFR ligand HB-EGF to transactivate EGFR, contributing to anti-apoptosis and maintenance of the epithelial barrier [20]. EGFR/Akt pathway activation is required for live LGG or LGG-derived soluble protein p40-stimulated suppression of apoptosis in a concentration-dependent manner [57]. Taken together, these results suggest that *L*. *rhamnosus*-mediated EGFR-independent Akt activation may favor IEC activation in response to bacterial infection.

In conclusion, our findings suggest that *L*. *rhamnosus* ATCC 7469 protects IPEC-J2 cells from F4^+^ ETEC infection, partly through reducing the adhesion of F4^+^ ETEC to the cells and subsequent attenuation of F4^+^ ETEC-induced mucin layer destruction and suppression of apoptosis of IPEC-J2 cells. Moreover, *L*. *rhamnosus* promotes EGFR-independent Akt activation, which may promote activation of IPEC-J2 cells in response to bacterial infection, in turn increasing TJ integrity to optimize the barrier function and restrict pathogen invasion.
